# Membrane-Associated Guanylate Kinase Inverted 2 Regulates the Organization of Podocyte Actin Cytoskeleton through Its Interaction with *α*-Actinin-4 and Synaptopodin

**DOI:** 10.34067/KID.0000001034

**Published:** 2025-11-03

**Authors:** Mariko Ida, Hiroyuki Yamada, Naritoshi Shirata, Shin-ichi Makino, Koichiro Ichimura, Takayuki Miyaki, Issei Okunaga, Kaho Yamasaki, Yasuhiro Yoshimura, Hideki Yokoi, Masashi Mukoyama, Chiaki Iwamura, Kiyoshi Hirahara, Atsuhiro Taguchi, Katsuhiko Asanuma

**Affiliations:** 1 Department of Nephrology, Chiba University Graduate School of Medicine, Chiba, Japan; 2 The Laboratory for Kidney Research (TMK Project), Medical Innovation Center, Graduate School of Medicine, Kyoto University, Kyoto, Japan; 3 Department of Nephrology, Graduate School of Medicine, Kyoto University, Kyoto, Japan; 4 Department of Primary Care and Emergency Medicine, Graduate School of Medicine, Kyoto University, Kyoto, Japan; 5 Research Division, Mitsubishi Tanabe Pharmaceutical Corporation, Kanagawa, Japan; 6 Department of Anatomy and Life Structure, Juntendo University Graduate School of Medicine, Tokyo, Japan; 7 Laboratory of Morphology and Image Analysis, Research Core Facilities, Juntendo University Graduate School of Medicine, Tokyo, Japan; 8 Department of Nephrology, Kumamoto University Graduate School of Medical Sciences, Kumamoto, Japan; 9 Department of Immunology, Graduate School of Medicine, Chiba University, Chiba, Japan

**Keywords:** cell biology and structure, nephrology, podocyte, proteinuria

## Abstract

**Key Points:**

- The loss of membrane-associated guanylate kinase inverted 2 (MAGI-2) induces actin cytoskeleton reorganization in mice.
- MAGI-2, *α*-actinin-4, and synaptopodin form a complex *in vitro*.
- Regulating MAGI-2 has the potential to suppress foot process effacement and is a promising target for podocyte damage.

**Background:**

Podocytes form interdigitating foot processes connected by the slit diaphragm (SD), which serves as a size-selective and charge-selective barrier essential for glomerular filtration. In kidney diseases, it is known that podocyte injury leads to the reorganization of the actin cytoskeleton, resulting in foot process effacement, which contributes to proteinuria and the progression of the disease. However, the detailed mechanisms underlying the regulation of actin cytoskeleton reorganization remain unclear. This study aims to elucidate the role of the scaffolding protein membrane-associated guanylate kinase inverted 2 (MAGI-2) in actin cytoskeleton regulation and to explore its potential as a novel therapeutic target.

**Methods:**

MAGI-2 was specifically knocked out in podocytes, and the localization changes of the actin cytoskeleton were observed in mice. In addition, immunoprecipitation experiments were performed in cultured cells to identify the binding domains of MAGI-2 with *α*-actinin-4 and synaptopodin (Synpo), key components of the actin cytoskeleton, and their colocalization changes were analyzed.

**Results:**

In the absence of MAGI-2 in podocytes, actin bundles normally located in the center of the foot processes were found to shift toward the basement membrane, indicating that MAGI-2 contributes to cytoskeletal reorganization. Immunoprecipitation revealed that MAGI-2 forms a complex with Synpo and *α*-actinin-4, and co-expression in cultured podocytes induced a relocalization of the actin cytoskeleton toward cell-cell contact. These findings suggest that MAGI-2, in addition to its previously known role as a scaffolding protein of the SD, directly interacts with actin-associated proteins and plays a critical role in the reorganization of the podocyte cytoskeleton.

**Conclusions:**

MAGI-2 binds to Synpo and *α*-actinin-4, regulating actin filament localization and contributing to cytoskeletal reorganization in podocytes. These findings uncover a new molecular link between SD components and the actin cytoskeleton, offering insights into mechanisms of podocyte injury.

## Visual Abstract

**Figure:**
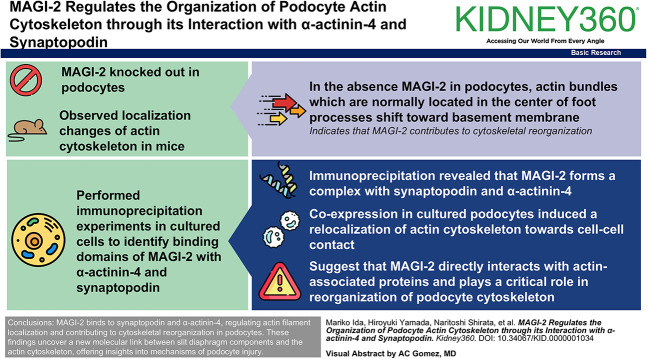

## Introduction

Podocytes are highly differentiated glomerular epithelial cells characterized by a unique morphological structure consisting of a cell body, primary processes, and foot processes. These foot processes interdigitate with those of the adjacent podocytes, covering the outer surface of the glomerular basement membrane (GBM). The slit diaphragm (SD), located between the interdigitating foot processes, functions as a size-selective and charge-selective barrier that plays a critical role in preventing protein leakage into urine.^1,2^ The foot processes of podocytes contain actin-rich cytoskeletal structures that dynamically respond to mechanical forces such as intracapillary pressure. Recent studies have suggested that, rather than containing classical contractile actin fibers, the foot processes of podocytes undergo continuous cytoskeletal remodeling, enabling adaptive changes in cell shape and adhesion. This dynamic regulation of the actin network is believed to contribute to the maintenance of the filtration barrier through mechanisms, such as mechanotransduction and cytoskeletal tensegrity.^3–6^ At sites of foot process effacement, thick actin bundles become redistributed from the central region of the foot process toward the basal area adjacent to the GBM.^7^ This redistribution reflects cytoskeletal reorganization associated with morphological changes in podocytes and disruption of the filtration barrier. Persistent podocyte damage may lead to apoptosis and, under certain pathological conditions, subsequently result in detachment from the GBM.^8^ This podocyte loss, coupled with ongoing proteinuria, contributes to glomerulosclerosis and the progression to CKD. Therefore, elucidating the mechanisms that regulating the actin cytoskeleton in podocytes is crucial for preventing CKD progression.

The cell body and primary processes of podocytes are composed of intermediate filaments, whereas the foot processes are composed of actin.^3,4^ The thick actin bundles in the central region of the foot processes consist of F-actin bundled by *α*-actinin-4.^9,10^ Mutations or deletions of *α*-actinin-4 are known to cause foot process abnormalities,^11–14^ highlighting their critical role in maintaining the foot process structure. Synaptopodin (Synpo) is also localized within the actin bundles of foot processes and interacts with *α*-actinin-4 to promote the formation of actin filaments in podocytes.^15^ Emerging evidence indicates Synpo is closely associated with the Rho family of small GTPases, which play an essential role in regulating the actin cytoskeleton. In cultured podocytes, Synpo has been shown to cooperate with Ras homolog family member A (RhoA) to induce the formation of actin stress fibers.^16^ In addition, Synpo binds to IRSp53, thereby inhibiting the assembly of the IRSp53-Mena-Cdc42 complex, which promotes filopodia formation.^17^ Recent research further indicates that Synpo regulates actin dynamics in podocytes by enhancing the binding of *α*-actinin-4 to actin and modulating the balance between active RhoA and Rac1.^18,19^ Although *α*-actinin-4 and Synpo are known to be involved in the formation and maintenance of the actin cytoskeleton of the foot process, their precise regulatory mechanisms remain unknown.

Membrane-associated guanylate kinase inverted 2 (MAGI-2) is a member of the membrane-associated guanylate kinase (GuK) protein family. Membrane-associated GuK proteins are known to localize to cell–cell contact regions, such as tight junctions in epithelial cells and synaptic connections in neurons.^9,20^

MAGI-2 is crucial for maintaining SD structure and podocyte survival. MAGI-2 knockout (KO) mice are anuric and succumb to renal failure within 24 hours of birth.^21^ Studies on MAGI-2 KO mice have demonstrated that the loss of MAGI-2 leads to increased expression of cathepsin L, a protein essential for the rearrangement of the actin cytoskeleton in podocytes, as well as abnormal expression of various adhesion-related molecules.^21^

Our laboratory recently developed podocyte-specific MAGI-2 KO mice and conducted studies on the role of MAGI-2 in maintaining SD structure. We have demonstrated that loss of MAGI-2 leads to nuclear translocation of dendrin, promoting podocyte apoptosis.^22^ In addition, we have shown that MAGI-2 is involved in the localization and stabilization of nephrin and neph1, which are major components of the SD, thereby contributing to the maintenance of its structure.^23^ Although these findings suggest that MAGI-2 is a critical scaffold protein for proper SD formation, its role in regulating the podocyte actin cytoskeleton remains poorly understood. Notably, MAGI-2 contains multiple protein–protein interaction domains (*e.g*., postsynaptic density 95/discs large/zonula occludens-1 (PDZ) and WW), suggesting a potential role in linking SD proteins to actin-associated cytoskeletal components.

In this study, we demonstrated for the first time that MAGI-2 forms a molecular complex with *α*-actinin-4 and Synpo, two key regulators of the actin cytoskeleton in podocytes. Our findings highlighted a previously unrecognized molecular link between the SD-associated protein MAGI-2 and actin-binding proteins, suggesting a structural basis for the coordination between SD components and the cytoskeleton.

## Methods

See Supplemental Materials and Methods for detailed descriptions.

### Immunofluorescence Analysis

Immunofluorescence (IF) analyses of mouse frozen kidney sections and cultured podocytes were performed as previously described.^24^ IF images were obtained using a Zeiss LSM780 (Zeiss, Oberkochen, Germany) and Nikon structured illumination microscopy (Nikon Instruments Inc., Tokyo, Japan). Mice kidney sections were labeled with anti-MAGI-2 (M2441, rabbit, 1:100; Sigma-Aldrich), anti-Synpo (65,194, mouse, 1:10; PROGEN), anti-*α*-actinin-4 (0042-05, rabbit, 1:200; immunoGrobe), Rhodamine Phalloidin (R415, Invitrogen), Podocalyxin (AF1556-SP, goat, 1:70; R&D Systems), Nidogen (ab14511, rabbit, 1:200; abcam), Nephrin (AF3159, goat, 1:100; R&D Systems), and zonula occludens-1 (ZO-1) (sc33725, mouse, 1:50; Santa Cruz Biotechnology). For IF analysis and quantification, kidney sections were obtained from four control and four tamoxifen inducible podocyte-specific MAGI-2 KO (MAGI-2 Ipd KO) mice at 4 weeks after tamoxifen injection.

### Electron Microscopy

The kidneys were fixed in 2.5% glutaraldehyde/0.1 M phosphate buffer. Fixed kidney samples were processed by multiple *en bloc* staining^25^ to obtain a higher image contrast. Ultrathin sections were prepared using a histo Jumbo diamond knife (DiATOME, Nidau, Switzerland) and collected on a silicon wafer piece. After electron staining with a 2% aqueous solution of gadolinium acetate and Reynolds's lead solution, the sections were imaged using a backscatter electron detector on a JSM-IT800 field-emission scanning electron microscopy (JEOL, Tokyo, Japan), as described previously.^26^ Foot process width was assessed at 20 randomly selected sites from three glomeruli each, in representative control and MAGI-2 Ipd KO mice.

### Human Kidney Biopsy Samples

Human kidney samples were obtained from six individuals: three with minor glomerular abnormalities (MGA) and three with FSGS. Among the MGA samples, two were obtained from diagnostic renal biopsies, and one was derived from the normal portion of a nephrectomized kidney resected for renal cell carcinoma. All samples were collected at the Chiba University Hospital. Tissues were fixed in 100% cold acetone and processed for IF analysis. This study was conducted in accordance with the Declaration of Helsinki, with written informed consent obtained from all participants, and was approved by the Ethics Committee on Human Research of Chiba University Hospital (Reference No. 1178).

### Spatial Analysis of Synpo, Podocalyxin, and Nidogen Signals in Mouse Glomeruli

To evaluate the spatial relationship between the actin-associated protein Synpo (green), apical membrane marker podocalyxin (magenta), and GBM marker nidogen (gray), the IF signals in mouse glomeruli were analyzed. Each optical section was examined to identify the capillary loops where the three markers delineated the tuft surface in an approximately parallel manner, indicating that the loops were sectioned nearly perpendicular to the imaging plane. Regions fulfilling these criteria were selected, and a line perpendicular to the alignment across the glomerular capillary loops was drawn. Fluorescence intensity profiles were generated using the profile plot function of Nikon imaging software elements (Nikon Corporation). The peak intensities of each marker were identified from the profiles. The distance from the Synpo peak to the nidogen peak was divided by the distance from the podocalyxin peak to the nidogen peak to calculate a positional ratio. This analysis was performed at 30 randomly selected measurement points from four control and four MAGI-2 Ipd KO mice (three glomeruli per mouse). Statistical comparisons were conducted using the Mann–Whitney *U* test. In this study, *n* refers to the number of line measurements (30 per mouse) derived from the glomeruli.

### Image Analysis

ImageJ software 1.54f (National Institutes of Health) was used to measure the intensity at cell–cell contact of MAGI-2 overexpressing podocytes (MAGI-2 OE podocytes). First, a two-dimensional graph of the luminance of pixels along a straight line in the image is prepared using the ImageJ plot profile. Then, the luminance and distance data were stored, and both red and green luminance and distance data were made into one graph using R version 4.1.2 (the R Foundation for Statistical Computing, Vienna, Austria). The Pearson correlation coefficient (R) was used to quantify membrane colocalization of Synpo and *α*-actinin-4. Data were analyzed using a *t* test.

### Statistical Analysis

All statistical analyses were performed using R version 4.1.2. Statistical significance between the two groups was evaluated using a *t* test and was determined to be statistically significant at *P* < 0.05. Data are represented as the mean±SEMs.

## Results

### Depletion of MAGI-2 in Adult Mouse Podocytes Induces Actin Cytoskeleton Reorganization

Previously, we generated podocyte-specific MAGI-2 KO mice by crossing floxed Magi2 and Nphs2-cre mice.^21,22^ Nphs2-Cre has been shown to excise floxed genes specifically in the podocytes of neonatal capillary loop-stage glomeruli.^27^ These mice develop significant albuminuria and segmental glomerulosclerosis at 8 weeks of age, progressing to global glomerulosclerosis by 12 weeks and ultimately leading to renal failure and death by 20 weeks.

**Figure 1:**
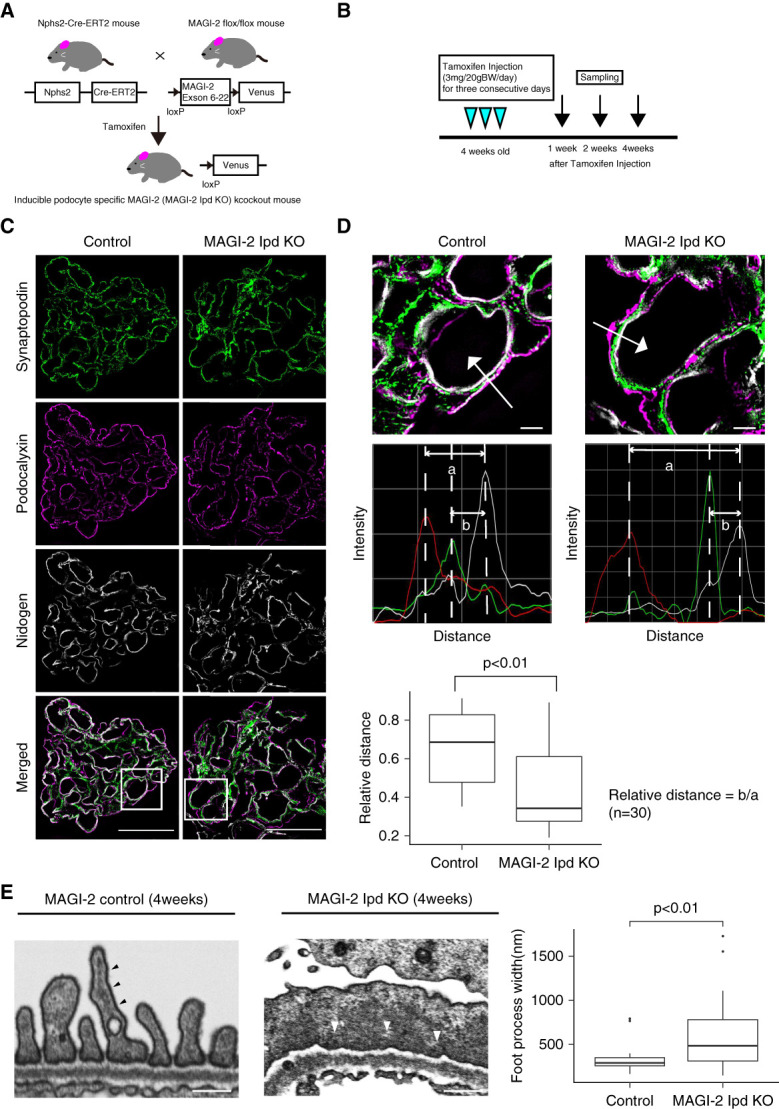
**Podocyte-specific deletion of MAGI-2 induces cytoskeletal disorganization and foot process effacement in mice.** (A) An overview of the breeding strategies used to generate MAGI-2 Ipd KO mice. (B) A schematic representation of the timeline for tamoxifen administration and subsequent kidney collection. (C) Representative images of Synpo (green), podocalyxin (magenta), and nidogen (gray) staining in control and MAGI-2 Ipd KO mice 4 weeks after tamoxifen injection. Bars=10 *μ*m. (D) The upper panel in (D) shows a higher magnification view of the area outlined by the white box in (C). Quantification of the spatial relationships among green-, magenta-, and gray-labeled signals was performed. Regions where the three signals were aligned in parallel were selected, and fluorescence intensity profiles were obtained using profile plots drawn perpendicular to the gray signal. The distance from the peak green intensity to the peak gray intensity (b) was divided by the distance from the peak magenta intensity to the peak gray intensity (a). This ratio was compared between control and MAGI-2 Ipd KO mice. Arrows indicate representative positions used for fluorescence intensity profiling. Thirty measurement points were randomly selected from four control and four MAGI-2 Ipd KO mice. Bars=1 *μ*m. (E) Transmission electron microscope images in control and MAGI-2 Ipd KO mice 4 weeks after tamoxifen injection. The arrowheads indicate regions of high electron density. Bars=1 *μ*m. Right: quantification of foot process width based on measurements from electron micrographs. Foot process width was significantly increased in MAGI-2 Ipd KO mice compared with controls. MAGI-2, membrane-associated guanylate kinase inverted 2; MAGI-2 Ipd KO, inducible podocyte-specific membrane-associated guanylate kinase inverted 2 knockout; Synpo, synaptopodin.

To analyze the role of MAGI-2 in mature adult podocytes, we used a tamoxifen-MAGI-2 Ipd KO strategy by crossing floxed-Magi2 and Nphs2-CreER^T2^ mice^23^ (Figure 1A). Tamoxifen injection at 4 weeks of age induced proteinuria at 20 weeks and glomerulosclerosis 6 months after tamoxifen administration.^23^ This relatively slow-progressing phenotype was considered suitable for studying temporal changes in the podocyte actin cytoskeleton.

To establish the spatial relationship between MAGI-2 and cytoskeletal components under physiological conditions, we performed triple IF staining on kidney sections from wild-type mice using combinations of MAGI-2, Synpo, *α*-actinin-4, and nephrin. These analyses revealed that MAGI-2 colocalized with both Synpo and *α*-actinin-4 in podocytes, supporting the idea that MAGI-2 is associated with the actin cytoskeleton close to the SD *in vivo* (Supplemental Figure 1).

First, we confirmed the timing of MAGI-2 depletion by harvesting kidneys at 1, 2, and 4 weeks after tamoxifen administration and performing immunohistochemistry (Figure 1B). As previously reported,^23^ MAGI-2 expression decreased 2 weeks after tamoxifen injection and was nearly absent in IF after 4 weeks of treatment (Supplemental Figures 2A and 3). Next, we examined the overall distribution of F-actin in the glomeruli to assess the cytoskeletal organization. Phalloidin staining of kidney sections from control and MAGI-2 Ipd KO mice at consecutive time points after tamoxifen administration revealed distinct differences (Supplemental Figure 2A). In the control mice, F-actin was predominantly localized in the mesangial region. By contrast, MAGI-2 Ipd KO mice showed increased F-actin signals not only in the mesangial region but also along the capillary loops. These findings indicate that the loss of MAGI-2 induces cytoskeletal reorganization involving both the mesangial and podocyte regions, prompting further analysis of podocyte-specific actin structures.

We then analyzed the distribution of Synpo and *α*-actinin-4, both of which are involved in forming actin bundles in podocytes. In control mice, Synpo and *α*-actinin-4 were broadly distributed throughout the cytoplasmic region of podocytes, whereas in MAGI-2 Ipd KO mice, their staining pattern shifted to align with the capillary loops (Supplemental Figure 2B). To investigate early cytoskeletal changes before overt proteinuria, which is typically observed approximately 20 weeks after tamoxifen injection,^23^ we performed super-resolution structured illumination microscopy (SIM) at 4 weeks after tamoxifen injection, a stage without overt proteinuria. We stained Synpo along with podocalyxin and nidogen to label the apical and GBMs, respectively. SIM imaging enabled detailed visualization of protein localization, and quantitative analysis revealed significant alterations in Synpo distribution relative to these markers in MAGI-2 Ipd KO mice compared with controls (Figure 1, C and D). Specifically, as shown in Figure 1D, we quantified the position of Synpo relative to apical and basal markers by calculating the ratio of the distance from nidogen to Synpo to the distance from nidogen to podocalyxin along the capillary loops. This approach allowed us to visualize and quantify the shift in Synpo localization within podocytes of MAGI-2 Ipd KO mice. These results support the conclusion that cytoskeletal rearrangement occurs early after MAGI-2 deletion. In addition, transmission electron microscopy analysis at the stage of 4 weeks after tamoxifen injection revealed the presence of foot process effacement in MAGI-2 Ipd KO mice compared with control mice, with electron-dense structures identified as actin filament bundles observed along the GBM (Figure 1E). Together, these findings indicate that a reorganization of the actin cytoskeleton components and foot process morphology occurring before the onset of significant proteinuria, which has been reported at 20 weeks post-KO,^23^ suggesting that these morphological changes represent an early event in disease progression.

Altered localization of actin-associated proteins has been previously reported in rats with puromycin aminonucleoside-induced nephropathy.^28–30^ In line with these findings, we observed similar changes in a mouse model of adriamycin (ADR) nephropathy. Fourteen days after ADR administration, MAGI-2 expression was reduced compared with control mice, and IF staining revealed that both Synpo and *α*-actinin-4 became predominantly localized along the glomerular capillary loops (Supplemental Figure 4A).

To assess the relevance to human disease, we analyzed renal tissues with minimal histological abnormalities obtained from kidney biopsies diagnosed as MGA and from nontumor areas of nephrectomy specimens. Consistent with our previous observations,^23^ MAGI-2 expression was lower in patients with FSGS than in those with MGA. In parallel, both Synpo and *α*-actinin-4 displayed a shift in localization from a broad intracellular distribution to a pattern concentrated along the glomerular capillary loops (Supplemental Figure 4B). These findings are parallel with those seen in MAGI-2 Ipd KO mice, indicating that decreased MAGI-2 expression in human glomerular disease is associated with altered localization of the actin-associated proteins Synpo and *α*-actinin-4. Next, we examined whether this shift in localization also reflected changes along the apicobasal axis and we performed triple immunostaining for podocalyxin, nidogen, and Synpo on human kidney biopsy specimens. SIM and quantitative analysis demonstrated that in FSGS samples, Synpo was positioned significantly closer to nidogen compared with MGA controls, indicating a redistribution of Synpo toward the basolateral, GBM-facing side of the podocytes (Supplemental Figure 2, C and D).

### MAGI-2, *α*-Actinin-4, and Synpo Form a Complex *In Vitro*

Previously, we demonstrated the role of MAGI-2 in controlling the localization of SD backbone proteins, where it interacts with Neph1 through the PDZ1 and PDZ5 domains and with nephrin through the PDZ3-5 domains in cultured podocytes.^23^ Another study has shown that MAGI-1 interacts with Synpo and *α*-actinin-4 in the human embryonic kidney cells.^31^ However, the role of MAGI-2 in actin cytoskeleton organization through interactions with Synpo or *α*-actinin-4 remains unclear.

Therefore, we conducted a coimmunoprecipitation (co-immunoprecipitation [IP]) experiment using human embryonic kidney T antigen cells cotransfected with green fluorescent protein (GFP)- or FLAG epitope tag (FLAG)-Synpo, *α*-actinin-4, and MAGI-2 (Supplemental Materials and Methods).

**Figure 2:**
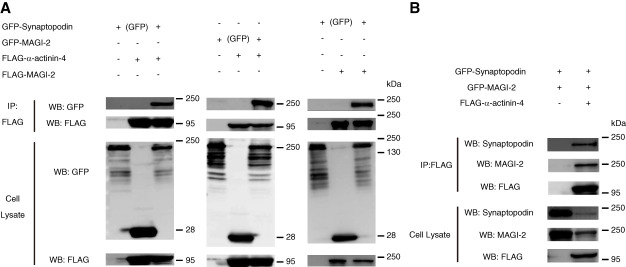
***α*-actinin-4, Synpo, and MAGI-2 bind each other.** (A) GFP-Synpo coprecipitated with FLAG-*α*-actinin-4 and FLAG-MAGI-2. GFP-MAGI-2 coprecipitated with FLAG-*α*actinin4. (B) GFP-Synpo, GFP-MAGI-2, and FLAG-*α*-actinin-4 were coprecipitated simultaneously. FLAG, FLAG epitope tag; GFP, green fluorescent protein; IP, immunoprecipitation; WB, Western blot.

GFP-Synpo coimmunoprecipitated with FLAG-*α*-actinin-4, confirming the interaction between Synpo and *α*-actinin-4 (Figure 2A, left panel). Similarly, GFP-MAGI-2 coimmunoprecipitated with FLAG-*α*-actinin-4 (Figure 2A, middle panel), and GFP-Synpo coimmunoprecipitated with FLAG-MAGI-2 (Figure 2A, right panel). These results demonstrate the interactions between MAGI-2 and *α*-actinin-4, and between Synpo and MAGI-2, respectively (Figure 2A and Supplemental Figure 8).

Furthermore, cotransfection of human embryonic kidney T antigen cells with GFP-Synpo, GFP-MAGI-2, and FLAG-*α*-actinin-4 confirmed the binding of all three proteins, suggesting that they form a complex (Figure 2B and Supplemental Figure 9).

### MAGI-2 Interact with Synpo through Its WW Domains and with *α*-Actinin-4 through the GuK Domain

Next, to identify the domains in MAGI-2 that interact with Synpo and *α*-actinin-4, we performed co-IP experiments using truncated mutants of MAGI-2. MAGI-2 is a scaffolding protein that contains three distinct protein–protein interaction domains. On the *N*-terminus, the PDZ0 domain, Src homology 3 or WWP repeating motif (WW) domain, and GuK domain exists, whereas on the C-terminus contains repeated PDZ 1–5.^32^

**Figure 3:**
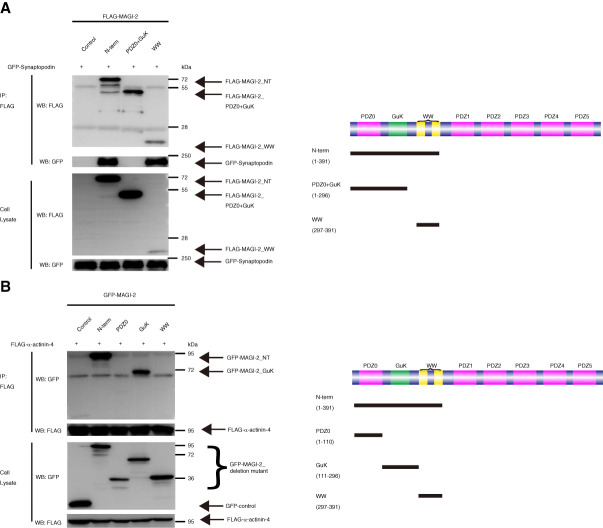
**Synpo binds the WW domain of MAGI-2, and *α*-actinin-4 binds the GuK domain of MAGI-2.** (A) GFP-Synpo coprecipitated with MAGI-2 *N*-terms and WW domains, but not PDZ0+GuK domains. The schematic of the plasmids used in the experiment is shown on the right. (B) FLAG-*α*-actinin-4 coprecipitated with MAGI-2 *N*-terms and GuK domains, but not PDZ0 and WW domains. The schematic of the plasmids used in the experiment is shown on the right. FLAG-MAGI-2 NT, FLAG-MAGI-2 *N*-term; GuK, guanylate kinase; PDZ, postsynaptic density 95/discs large/zonula occludens-1.

FLAG-MAGI-2 *N*-term and FLAG-MAGI-2 mutants containing WW domains interacted with GFP-Synpo, whereas FLAG-MAGI-2 mutants containing PDZ0 and GuK domains did not coprecipitated with GFP-Synpo. This indicates that MAGI-2 binds to Synpo through its WW domain (Figure 3A). Similarly, GFP-MAGI-2 *N*-term and GFP-MAGI-2 mutants containing GuK domains interacted with FLAG-*α*-actinin-4, while GFP-MAGI-2 mutants containing PDZ0 or WW domains did not. This indicates that MAGI-2 binds *α*-actinin-4 through the GuK domain (Figure 3B).

To examine the protein interactions involving MAGI-2, we performed glutathione S-transferase (GST) pull-down assays using glomerular lysates from mouse kidneys. Synpo was efficiently pulled down by GST-tagged MAGI-2, whereas *α*-actinin-4 was not detected (Supplemental Figure 5). By contrast, co-IP assays using overexpressed proteins demonstrated an interaction between MAGI-2 and *α*-actinin-4 (Figure 2).

### MAGI-2 Translocates Long Actin Bundles to the Cell–Cell Contact Regions in CV-1 in Origin, SV40-transformed African Green Monkey Kidney Fibroblast Cells

In the next step, to examine the combinatory roles of Synpo, *α*-actinin-4, and MAGI-2 in the cells, we introduced these genes in CV-1 in Origin, SV40-transformed African green monkey kidney fibroblast cells (COS7) cells in various combinations and examined their subcellular localization through immunofluorescent staining. To this end, we used COS7 cells because they lack the endogenous expression of these proteins and are highly amenable to transfection and immunostaining. These features enabled us to assess the localization and interaction of the introduced proteins without interference.

**Figure 4:**
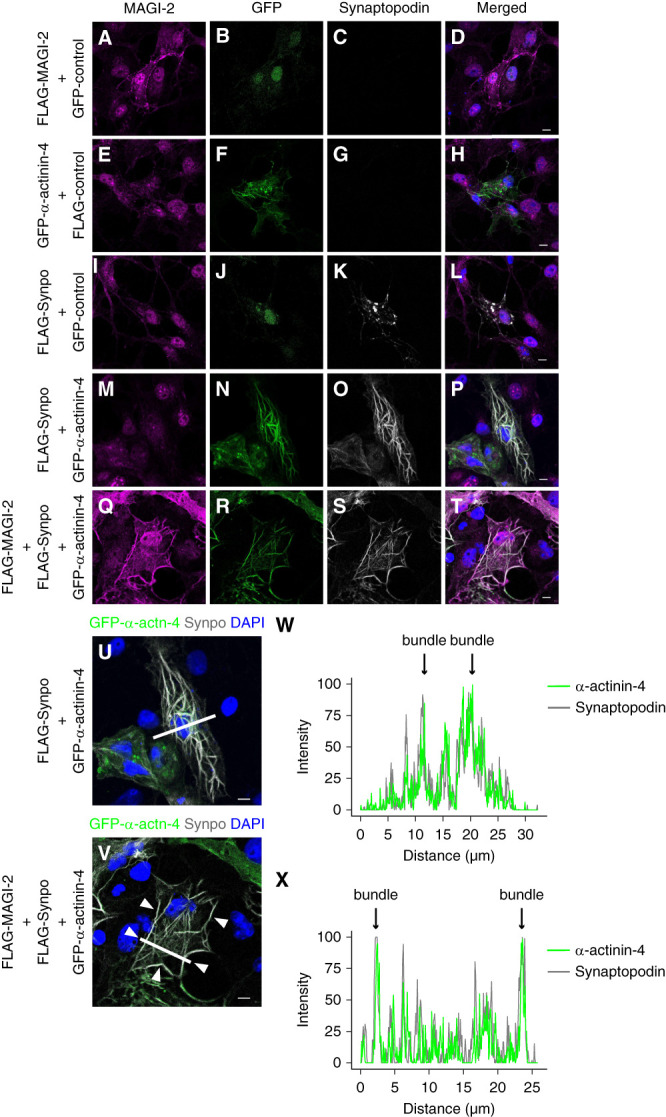
***α*-actinin-4, Synpo, MAGI-2 complex localize at cell-cell contact of COS7 cell.** Representative IF images (A–T) of COS7 cells transfected with FLAG-MAGI-2, GFP-*α*-actinin-4, and FLAG-Synpo. Panels (A–D) show cells transfected with FLAG-MAGI-2 and GFP-control: (A) stained for MAGI-2; (B) GFP fluorescence (GFP control); (C) stained for Synpo; (D) merged image. Panels (E–H) show cells transfected with GFP-*α*-actinin-4 and FLAG-control: (E) MAGI-2; (F) GFP-*α*-actinin-4; (G) Synpo; (H) merged. Panels (I–L) show cells transfected with FLAG-Synpo and GFP-control: (I) MAGI-2; (J) GFP-control; (K) Synpo; (L) merged. Panels (M–P) show cells transfected with FLAG-Synpo and GFP-*α*-actinin-4: (M) MAGI-2; (N) GFP-*α*-actinin-4; (O) Synpo; (P) merged. Panels (Q–T) show cells transfected with FLAG-MAGI-2, FLAG-Synpo and GFP-*α*-actinin-4: (Q) MAGI-2; (R) GFP-*α*-actinin-4; (S) Synpo; (T) merged. Panels (U) and (V) show higher magnification views of merged images shown in panels (N and O) and (R and S), respectively. Fluorescence intensity profiles along the white lines in the left panels (U and V) are shown in the corresponding right panels (W and X, respectively). Arrows indicate actin bundles formed at sites where *α*-actinin-4 (green) and Synpo (gray) colocalize. Bars=20 *μ*m. IF, immunofluorescence.

Transfection of Synpo resulted in the formation of cytoplasmic actin aggregates (Figure 4K), while transfection of *α*-actinin-4 resulted mainly in short, branched actin filaments and some long filaments (Figure 4F). Cotransfection of *α*-actinin-4 and Synpo transformed the short filaments into long actin bundles (Figure 4, U and W). The fluorescence intensity profiles revealed that both Synpo and *α*-actinin-4 exhibited a relatively dominant distributions in the cellular body region. By contrast, when FLAG-MAGI-2 was coexpressed with FLAG-Synpo and GFP–*α*-actinin-4, both of the Synpo and *α*-actinin-4 signals became more pronounced at the cell-cell contact (Figure 4, V and X and Supplemental Figure 6B). These data suggest that MAGI-2 forms a complex with *α*-actinin-4 and Synpo, facilitating the translocation of long actin bundles to cell–cell contact regions.

### MAGI-2 Translocates *α*-Actinin-4 and Synpo to the Cell–Cell Contact of MAGI-2 Overexpression Podocytes

To examine the roles of MAGI-2 and associated cytoskeletal proteins in a physiological context, we conducted experiments using cultured podocytes to determine whether the changes in protein localization observed in COS7 cells could be replicated. As previously reported,^23^ cultured podocytes do not express MAGI-2; therefore, we generated MAGI-2 OE podocytes by forced expression of MAGI-2 using piggyBac vectors.

To assess whether MAGI-2 overexpression affects the localization of actin fibers, we first performed co-IF staining for Synpo and F-actin (phalloidin) in cultured podocytes. In control podocytes, Synpo and F-actin colocalized primarily as stress fibers in the cell body (Supplemental Figure 7A, upper panels). By contrast, MAGI-2 overexpressing podocytes showed partial redistribution of Synpo and F-actin at cell–cell contact sites (Supplemental Figure 7A, lower panels).

**Figure 5:**
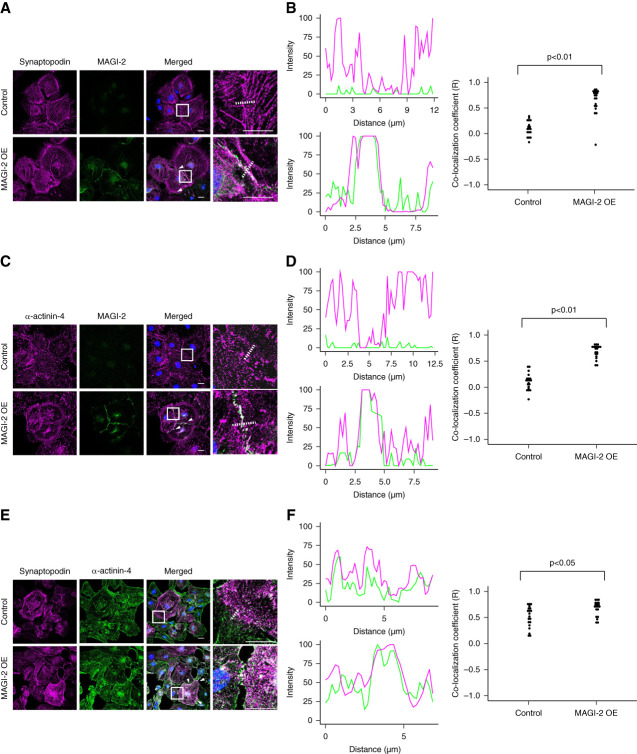
***α*-actinin-4, Synpo, MAGI-2 complex localize at cell–cell contact of MAGI-2 OE podocytes.** (A) Representative IF images showing Synpo, *α*-actinin-4, and MAGI-2 staining in control and MAGI-2 OE podocytes. The arrowheads indicate merged areas. The white boxes indicate regions displayed at higher magnification. Bars=20 *μ*m. (B) Cellular colocalization analysis of Synpo, *α*-actinin-4, and MAGI-2 in control and MAGI-2 OE podocytes. Fluorescence intensity profiles along the white lines in (A) are shown in the left panels. Pearson colocalization coefficient (R) was quantified for control and MAGI-2 OE podocytes, and the results are displayed in the right panels. *P* values were calculated using a *t* test (*n*=20). MAGI-2 OE podocytes, membrane-associated guanylate kinase inverted 2 overexpressing podocytes.

Next, podocytes were stained with Synpo and MAGI-2. As expected, MAGI-2 was not expressed in control podocytes but was present at cell–cell contacts in MAGI-2 OE podocytes (Figure 5A). Synpo was expressed as stress fibers in the cell bodies of both types of podocytes (Figure 5A). In MAGI-2 OE podocytes, Synpo was partially localized at cell–cell contacts and colocalized with MAGI-2 (Figure 5, A and B).

Similarly, *α*-actinin-4 was partially colocalized with MAGI-2 at cell–cell contacts in MAGI-2 OE podocytes (Figure 5, C and D).

Next, we costained control podocytes and MAGI-2 OE podocytes with Synpo and *α*-actinin-4. In both types of podocytes, Synpo and *α*-actinin-4 colocalized in the cell body, forming stress fibers. In MAGI-2 OE podocytes, Synpo and *α*-actinin-4 were partially colocalized at cell–cell contact regions (Figure 5, E and F).

To confirm that the redistribution of cytoskeletal molecules occurred at the sites of cell–cell contact, we performed IF staining for ZO-1, a well-established junctional marker. In control podocytes, Synpo and *α*-actinin-4 primarily formed stress fibers in the cell body and the colocalization with ZO-1 was limited (Supplemental Figure 7B, upper panels, and Supplemental Figure 7C, upper panels). By contrast, in MAGI-2-overexpressing podocytes, ZO-1 more frequently colocalized with Synpo or *α*-actinin-4, at cell–cell junctions (Supplemental Figure 7B, upper panels, and Supplemental Figure 7C, upper panels), indicating that these cytoskeletal proteins partly redistributed at cell-cell contact sites in the presence of MAGI-2.

Collectively, these results indicate that MAGI-2 drives the redistribution of *α*-actinin-4 and Synpo toward cell–cell contact sites by regulation of their subcellular localization in cultured podocytes.

Taken together, these findings establish MAGI-2 as a central regulator of cytoskeletal architecture that is essential for podocyte structure.

## Discussion

In this study, we report for the first time that MAGI-2 Ipd KO mice exhibit actin cytoskeletal reorganization. In addition, we demonstrate that MAGI-2, *α*-actinin-4, and Synpo interact and colocalize at cell–cell contacts in cultured podocytes.

In MAGI-2 Ipd KO mice, mislocalization of Synpo and *α*-actinin-4 precedes proteinuria, suggesting that cytoskeletal changes are an early events in podocyte injury leading to morphological and functional impairments. These alterations suggest that MAGI-2 regulates the actin cytoskeleton,^7,33^ which may act as a defense mechanism to prevent podocyte detachment from the GBM when podocytes are damaged.^34–36^ *In vitro* analyses revealed that MAGI-2 interacts with Synpo and *α*-actinin-4 through its WW and GuK domains, respectively, indicating direct binding between MAGI-2 and these cytoskeletal components. However, in GST pull-down assays using glomerular lysates, only Synpo was detected, whereas *α*-actinin-4 was not. This discrepancy suggests that the interaction between MAGI-2 and *α*-actinin-4 is transient, regulated, or dependent on the cellular context *in vivo*. Alternatively, other proteins may mediate or stabilize this interaction under physiological conditions.

In cultured podocytes overexpressing MAGI-2, Synpo and *α*-actinin 4 were partially localized to cell–cell contacts. In addition, while direct interactions between MAGI-2 and ZO-1 have not been reported, the observed changes in ZO-1 localization upon MAGI-2 overexpression suggest that MAGI-2 could influence ZO-1 indirectly, possibly through shared binding partners such as F-actin or *α*-actinin-4.^37–39^ Recent studies have indicated that MAGI-2 contributes to the upregulation of active RhoA expression.^40^ In addition, there is evidence linking MAGI-2 to Rap1, a small GTPase associated with the reorganization of the actin cytoskeleton.^41,42^ These findings suggest that MAGI-2 recruits Synpo and *α*-actinin-4 to cell–cell contacts and play a role in actin polymerization. Furthermore, Synpo is expressed at cell–cell contacts in nephrin stably expressing podocytes.^43^ CD2-associated protein (CD2AP) and cortactin, which stabilize actin nucleation and newly formed F-actin branches,^44^ are also localized at cell–cell contacts, indicating that molecules involved in actin reorganization are accumulated at cell-cell contacts in cultured podocytes.^45^ Synpo is known to bind CD2AP, and their combined heterozygosity has been associated with the development of FSGS.^46^ In addition, MAGI-2 forms a complex with *α*-actinin-4 and nephrin.^47^ Nephrin, in particular, has been implicated in actin cytoskeletal reorganization through its interactions with Nck and phosphoinositide 3-kinase.^48–50^ Integrating these research findings, MAGI-2 facilitates actin cytoskeleton reorganization by directing *α*-actinin-4 and Synpo to cell–cell junctions with nephrin and CD2AP.

Our research demonstrated that the loss of MAGI-2 *in vivo* results in actin cytoskeleton reorganization, whereas overexpression of MAGI-2 *in vitro* also induces changes indicative of actin cytoskeleton reorganization. One limitation of this study is that certain proteins essential for podocyte function *in vivo* are impaired in cultured podocytes, which limits the ability to replicate *in vivo* phenomena *in vitro* fully.^51^ The localization of MAGI-2, Synpo, and *α*-actinin-4 at cell–cell contacts likely indicates actin remodeling at the level of cultured cells. However, because these cell–cell contacts do not replicate SD or interdigitating structures, the extent to which these changes in cultured cells translate into morphological and functional alterations in foot processes *in vivo* remains uncertain. To overcome this limitation, kidney organoids, which allow three-dimensional culture, may provide a valuable tool for more accurate modeling. Another limitation is that our data could not clearly discriminate whether the positional changes of basal and apical markers reflect a specific cytoskeletal remodeling process or a more uniform morphological response to podocyte injury. Although similar positional shifts were observed in human biopsy samples, we cannot exclude the possibility that these reflect a general response to podocyte injury, rather than a specific mechanism that should be considered when interpreting human data. Finally, we cannot completely exclude the possibility that the observed effects were partially influenced by the presence of an artificial peptide such as the FLAG tag. In addition, the overexpression of multiple tagged proteins by transient transfection may have caused imbalanced expression levels, which could potentially affect their localization or interactions. This technical limitation has been acknowledged and should be considered when interpreting our *in vitro* results. On the basis of the hypothesis that actin cytoskeleton reorganization serves as a defense mechanism *in vivo*, we propose that MAGI-2 facilitates the translocation of Synpo and *α*-actinin-4 to the area above the GBM at an early stage of podocyte injury, thereby protecting the remaining nephrons.

Recently, mutations in MAGI-2 have been identified as one of the causes of steroid-resistant nephrotic syndrome, highlighting its crucial role in maintaining the structure and function of the SD.^40,52^ MAGI-2 is known to form a multiprotein complex with nephrin, IQ motif–containing GTPase-activating protein 1, calcium/calmodulin-dependent serine protein kinase, spectrin, and *α*-actinin-4.^47^ In addition, MAGI-2 has been shown to bind to *β*-catenin, which forms focal adhesions together with vinculin and paxillin.^53,54^ In this study, we demonstrated that MAGI-2 interacts with Synpo and *α*-actinin-4. These findings suggest that MAGI-2 functions as a linker connecting the SD, the actin cytoskeleton, and focal adhesions, thereby contributing to the structural and functional maintenance of podocyte foot processes. Furthermore, MAGI-2 seems to play a role in the regulating of signal transduction pathways. Previous findings from our laboratory demonstrated that MAGI-2 interacts with dendrin and regulates its localization in podocytes.^22^ More recently, we reported that nuclear translocation of dendrin promotes apoptosis in podocytes,^55^ suggesting that MAGI-2-mediated management of dendrin localization may be involved in podocyte injury mechanisms. In addition, recent studies have demonstrated reduced MAGI-2 expression in podocytes from patients with diabetic kidney disease, suggesting that MAGI-2 contributes to the maintenance of podocyte survival and function, potentially through the TGF-*β*1/Smad3 and TGF-*β*1/nephrin signaling pathways.^56^ In the brain, where research on MAGI-2 is advancing, numerous studies have reported on its role at neuronal junctions. MAGI-2 is involved in remodeling the postsynaptic cytoskeleton and contributes to synaptic plasticity and memory formation.^57^ In addition, MAGI-2 functions as a linker protein, forming protein complexes that facilitate signal transduction and help maintain the balance between excitatory and inhibitory synapses in neurons.^58–62^ On the basis of these findings, the specific role of MAGI-2 in the podocyte region remains largely unknown, highlighting the need for further *in vitro* functional analyses to clarify how MAGI-2 influences podocyte function and its underlying pathways. Although our findings provide structural and interactional insights into MAGI-2 in podocytes, the upstream regulatory mechanisms governing its expression and function remain to be elucidated. In ongoing studies, we are investigating candidate upstream regulators of MAGI-2 and their potential roles in modulating MAGI-2–mediated signaling pathways. These future efforts will be critical for understanding the broader regulatory network through which MAGI-2 contributes to podocyte homeostasis and the injury response.

Taken together, our findings support the hypothesis that MAGI-2 plays a crucial role in regulating the actin cytoskeleton in podocytes. Although further studies are needed to elucidate the precise regulatory mechanisms, the effective modulation of MAGI-2 has the potential to preserve cytoskeletal integrity and, ultimately, prevent the progression of CKD.

## Supplementary Material

## Data Availability

Original data generated for the study will be made available upon reasonable request to the corresponding author. Data Type: Raw Data/Source Data and Image Data.

## Declarative Statements

This study includes clinical experimentation and received Institutional Review Board or Ethics Committee approval. All patients provided written informed consent. This study includes clinical experimentation and complies with the Declaration of Helsinki. All animal experiments were conducted in accordance with the NIH Guide for the Care and Use of Laboratory Animals or an equivalent standard that meets or exceeds the ethical and welfare requirements outlined in the NIH Guide. All protocols were approved by the appropriate institutional animal care and use committee.

## Supplemental Material

Supplemental Materials and Methods

Supplemental Figure 1. Triple IF staining in wild-type mouse glomeruli to examine the localization of MAGI-2 and cytoskeletal proteins relative to nephrin.

Supplemental Figure 2. Altered localization of cytoskeletal and SD proteins in MAGI-2 Ipd KO mice and human glomerular diseases.

Supplemental Figure 3. Quantification of MAGI-2 fluorescence intensity in mouse glomeruli.

Supplemental Figure 4. Representative images of MAGI-2 and cytoskeletal protein localization in ADR nephropathy mice and human kidney samples.

Supplemental Figure 5. GST pull-down assay using mouse glomerular lysates.

Supplemental Figure 6. Quantification of fluorescence intensity in COS7 cells.

Supplemental Figure 7. Synpo and *α*-actinin-4 with the cell–cell contact marker ZO-1 in control and MAGI-2 OE podocytes.

Supplemental Figure 8. Full-length Western blot membranes for co-IP (Figure 2A).

Supplemental Figure 9. Full-length Western blot membranes for co-IP (Figure 2B).
