# Supplementary material for: Membrane-Associated Guanylate Kinase Inverted 2 Regulates the Organization of Podocyte Actin Cytoskeleton through Its Interaction with α-Actinin-4 and Synaptopodin
Source: Kidney360. 2025 Nov 3;7(2):247–59. doi: 10.34067/KID.0000001034 (PMC12935370; doi:10.34067/KID.0000001034)
Supplement: Supplementary file 2 [file kidney360-7-247-s002.pdf]

Supplemental Material

Table of Contents

Materials and Methods 3-7

Supplemental Figure 1. Triple immunofluorescence staining in wild-type mouse glomeruli to examine the localization of MAGI-2 and cytoskeletal proteins relative to nephrin 8

Supplemental Figure 2. Altered localization of cytoskeletal and slit diaphragm proteins in inducible podocyte-specific MAGI-2 knockout mice and human glomerular diseases 9

Supplemental Figure 3. Quantification of MAGI-2 fluorescence intensity in mouse glomeruli 10

Supplemental Figure 4. Representative images of MAGI-2 and cytoskeletal protein localization in ADR nephropathy mice and human kidney samples 11

Supplemental Figure 5. GST pull-down assay using mouse glomerular lysates 12

Supplemental Figure 6. Quantification of fluorescence intensity in COS7 cells 13

Supplemental Figure 7. Synaptopodin and α-actinin-4 with the cell–cell contact marker ZO-1 in control and MAGI-2 OE podocytes 14

Supplemental Figure 8. Full-length western blot membranes for co-immunoprecipitation (Figure 2A) 15

Supplemental Figure 9. Full-length western blot membranes for co-immunoprecipitation (Figure 2B) 16

Supplemental Figure Legends..17-22

Materials and Methods

Generation of podocyte-specific MAGI-2 knock-out mice

Podocyte-specific MAGI-2 knock-out (MAGI-2 pdKO) mice, which lacked MAGI-2 exons 6–22, were generated as previously described.[21, 22] Tamoxifen-inducible acquired podocyte-specific MAGI-2 knockout (MAGI-2 IpdKO) mice were also generated as previously described.[27] MAGI-2 deletion was induced by tamoxifen intraperitoneal injection (3mg/20gBW/day) for three consecutive days at the age of 4 weeks. Tamoxifen was dissolved in 100 μl ethanol and diluted to 15 mg/mL with autoclaved sunflower oil.

Cell culture and transient transfection

Conditionally immortalized mouse podocytes were cultured as described previously.[28] Transient transfections to HEK293T cells and COS7 were done as previously explained.[21]

Plasmid constructs

A full-length cDNA clone of mouse MAGI-2 was obtained from GenScript (Piscataway, NJ) and cloned in-frame into pFLAG-CMV-6a or pEGFP-C1(Sigma-Aldrich, St. Louis, MO) vectors. The N-term included PDZ0, GuK, and WW domains (amino acids 1-391), PDZ0 domain (amino acids 1-110), GuK domain (amino acids 111-296), PDZ0+GuK domains (amino acids 1-296), WW domain (amino acids 297-

391) and fragments of mouse MAGI-2 were generated by PCR and cloned into either pFLAG-CMV-6a or pEGFP-C1. Plasmids containing α-actinin-4 were provided by M. Pollak (Brigham and Women's Hospital, Boston, Massachusetts, USA)[14]. Empty vectors (pEGFP and pFLAG) were used as a negative control. All constructs were verified by Sanger sequencing.

Immunoprecipitation

Co-IP and western blotting from transfected HEK293T cells were performed as previously described.[21] Briefly, GFP- and FLAG-tagged cDNA constructs were transfected into HEK293T cells using FuGENE 6 (Promega, Madison, WI). After 48 hours, cells were lysed with IP buffer containing protease inhibitor (Complete Mini) with or without phosphatase inhibitor (PhosSTOP). Lysates were then incubated for 3 hours with anti-FLAG beads (Wako, Osaka, Japan). The beads were washed five times with IP buffer to remove unbound proteins. Bound proteins were eluted with 4× Laemmli buffer/2-mercaptoethanol. Elutes, along with whole-cell lysates (serving as input), were analyzed by western blotting. Blots were probed with anti-FLAG (F1804, rabbit, 1:10,000; Sigma-Aldrich) and anti-GFP (598, rabbit, 1:1000; MBL) antibodies.

Establishment of a MAGI-2 overexpression podocyte cell line

MAGI-2 overexpressing podocytes cell line was established as described previously.[23] Briefly, the GFP sequence was removed from the pPB-EF1α-EiP-A piggyBac vector, and Flag-tagged MAGI-2 cDNA was cloned to the vector (pPB-EF1α-Flag-MAGI-2-iP-A). A control podocyte, pPB-EF1α-Control-iP-A, was constructed using inverse PCR. These piggyBac vectors and pHL-EF1a-hcPBase (piggyBac transposed

expression vector) were co-transfected into undifferentiated cultured podocytes via lipofection. After the transfection, a puromycin selection (1.25 μg/mL) was applied, and the remaining puromycin-resistant colonies were dissociated into single cells.

Fluorescence Intensity Quantification in Mouse Kidney Sections

Fluorescence intensity in mouse kidney sections was quantified using ImageJ software 1.54f (National Institutes of Health). For each glomerulus, the peripheral region was manually outlined based on the green fluorescence signal (MAGI-2), and the mean fluorescence intensity within this area was measured. Background fluorescence was determined by measuring signal intensity in adjacent non-glomerular regions, and this value was subtracted from the glomerular intensity. Quantification was performed in four control and four knockout mice, using three glomeruli randomly selected per mouse. Statistical comparisons were conducted using the Mann-Whitney U test.

Quantification of fluorescence intensity profiles in COS7 cells

COS7 cells were transfected with expression vectors encoding FLAG-synaptopodin and GFP–α-actinin-4, or with FLAG-MAGI-2, FLAG-synaptopodin, and GFP–α-actinin-4. After 48h, cells were fixed and imaged under identical exposure settings. For each condition, 10 random regions were selected, and fluorescence intensity profiles were generated using the *Plot Profile* function in ImageJ. In each profile, the outermost 5 μm region was defined as the cell–cell contact area, and the remaining portion as the cell body. The mean gray value per micrometer was calculated for both regions. Differences between the two regions were analyzed using the Wilcoxon rank-sum test.

Fluorescence intensity and colocalization analysis in cultured podocytes

Cultured podocytes were transduced with control or MAGI-2 overexpression (OE) constructs. For each condition, 10 random regions were selected, and a line was drawn across the ZO-1–positive cell–cell junction (indicated by white lines in Figure S7). Fluorescence intensity profiles along these lines were obtained using the *Plot Profile* function in ImageJ. The resulting data were analyzed in R to calculate Pearson's correlation coefficients (R) between ZO-1 and synaptopodin, or between ZO-1 and α-actinin-4. Statistical analysis was performed using the *t*-test.

Establishment of ADR Nephropathy Mouse Model

ADR nephropathy was established in 8-week-old male BALB/c mice (weighing 20–25 g) through a single intravenous administration of doxorubicin hydrochloride (11.5 mg/kg; Wako, Osaka, Japan), following a previously described protocol.[23]For immunofluorescence staining, three mice each from the vehicle-treated group and from the ADR group at day 14 post-injection were used.

GST pull-down assays

GST pull-down assays using GST–MAGI2 fusion protein and mouse glomerular lysates were conducted as previously reported[23], with minor modifications. Glomeruli were lysed in a buffer containing protease inhibitors (Complete Mini) and phosphatase inhibitors (PhosSTOP). GST-tagged MAGI2 was expressed in *E. coli* BL21 and immobilized on GST-agarose beads (GE Healthcare). The beads were

washed three times with assay buffer (PBS containing 1% Triton X-100), then incubated with the glomerular lysates for 90 minutes at 4°C under gentle rotation. After incubation, the beads were washed three times with lysis buffer, and bound proteins were eluted using Laemmli buffer containing 2-mercaptoethanol. Eluates, along with glomerular lysates as input controls, were analyzed by western blotting using anti-synaptopodin and anti-α-actinin-4 antibodies.

Figure S1

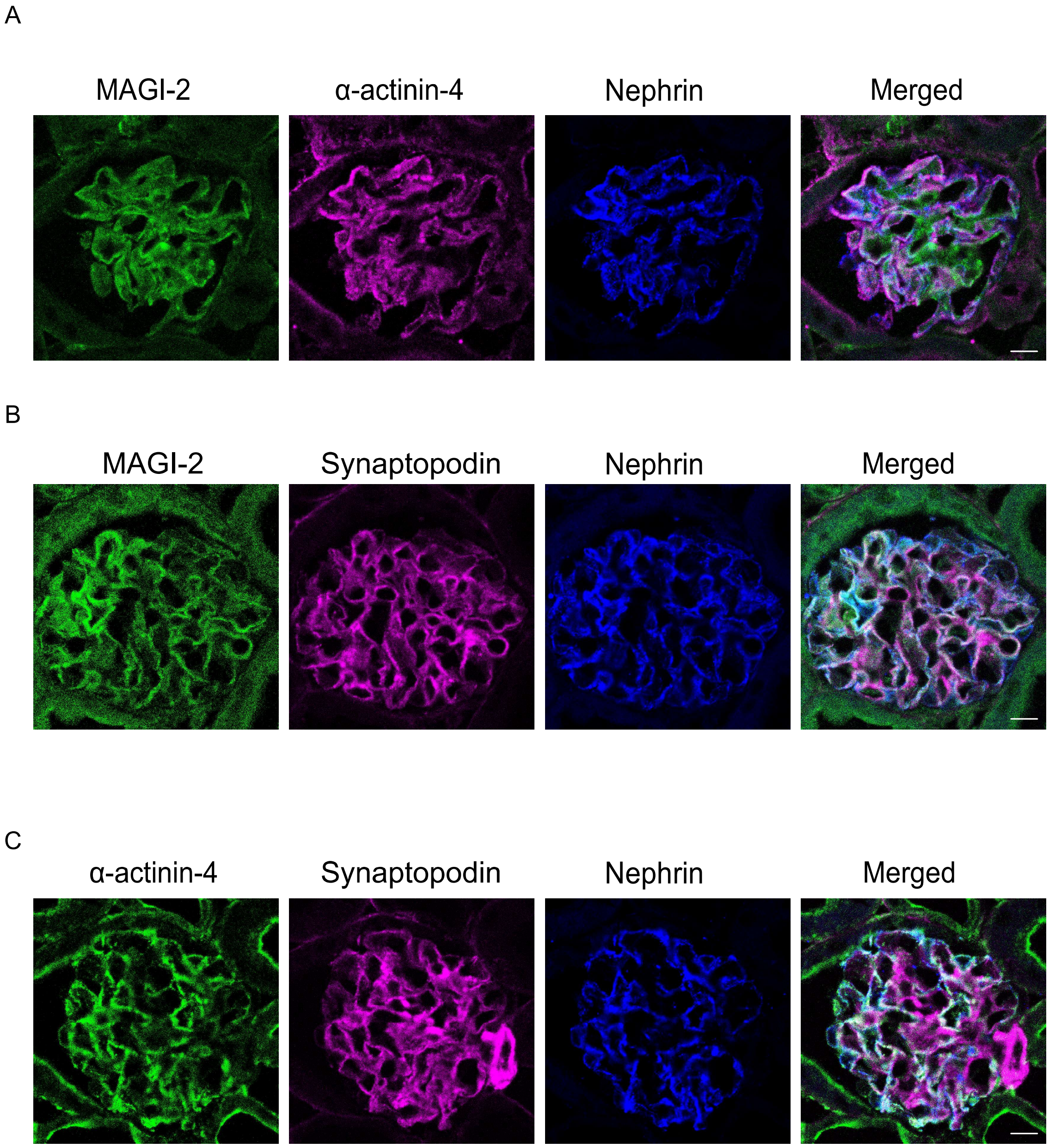
A
MAGI-2
α-actinin-4
Nephrin
Merged
B
MAGI-2
Synaptopodin
Nephrin
Merged
C
α-actinin-4
Synaptopodin
Nephrin
Merged

Figure S2

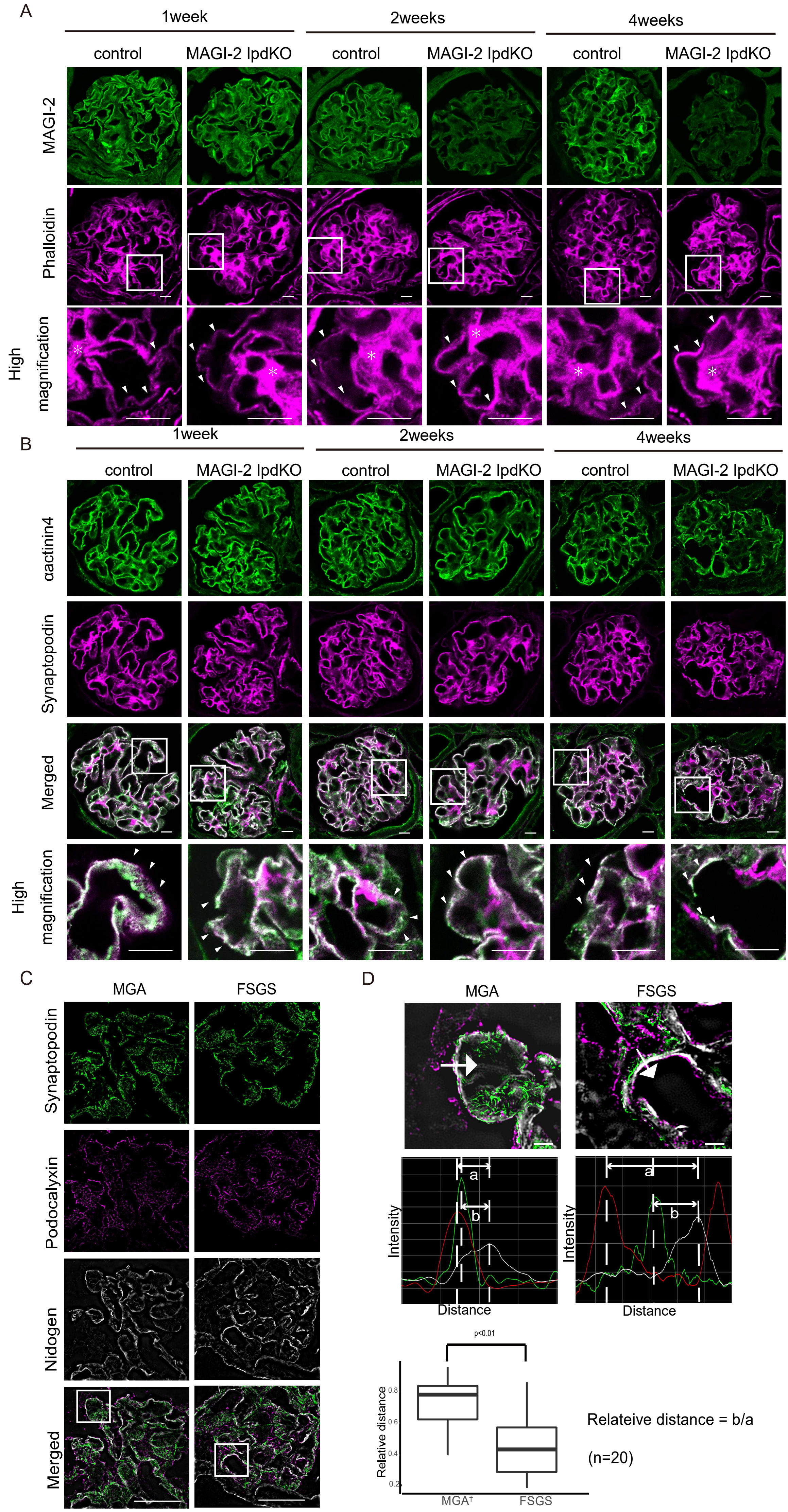
A
1week
2weeks
4weeks
control
MAGI-2 lpdKO
MAGI-2
Phalloidin
High magnification
B
αactinin4
Synaptopodin
Merged
C
MGA
FSGS
Podocalyxin
Nidogen
D
a
b
Intensity
Distance
p<0.01
Relative distance
MGA†
Relateive distance = b/a
(n=20)

Figure S3

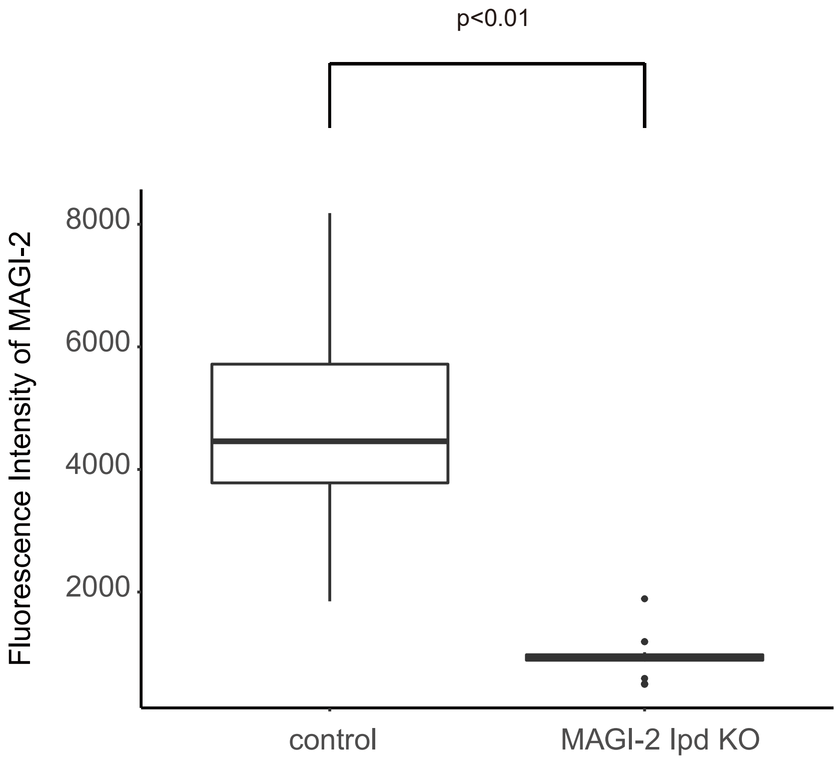
p<0.01
Fluorescence Intensity of MAGI-2
8000
6000
4000
2000
control
MAGI-2 Ipd KO

Figure S4

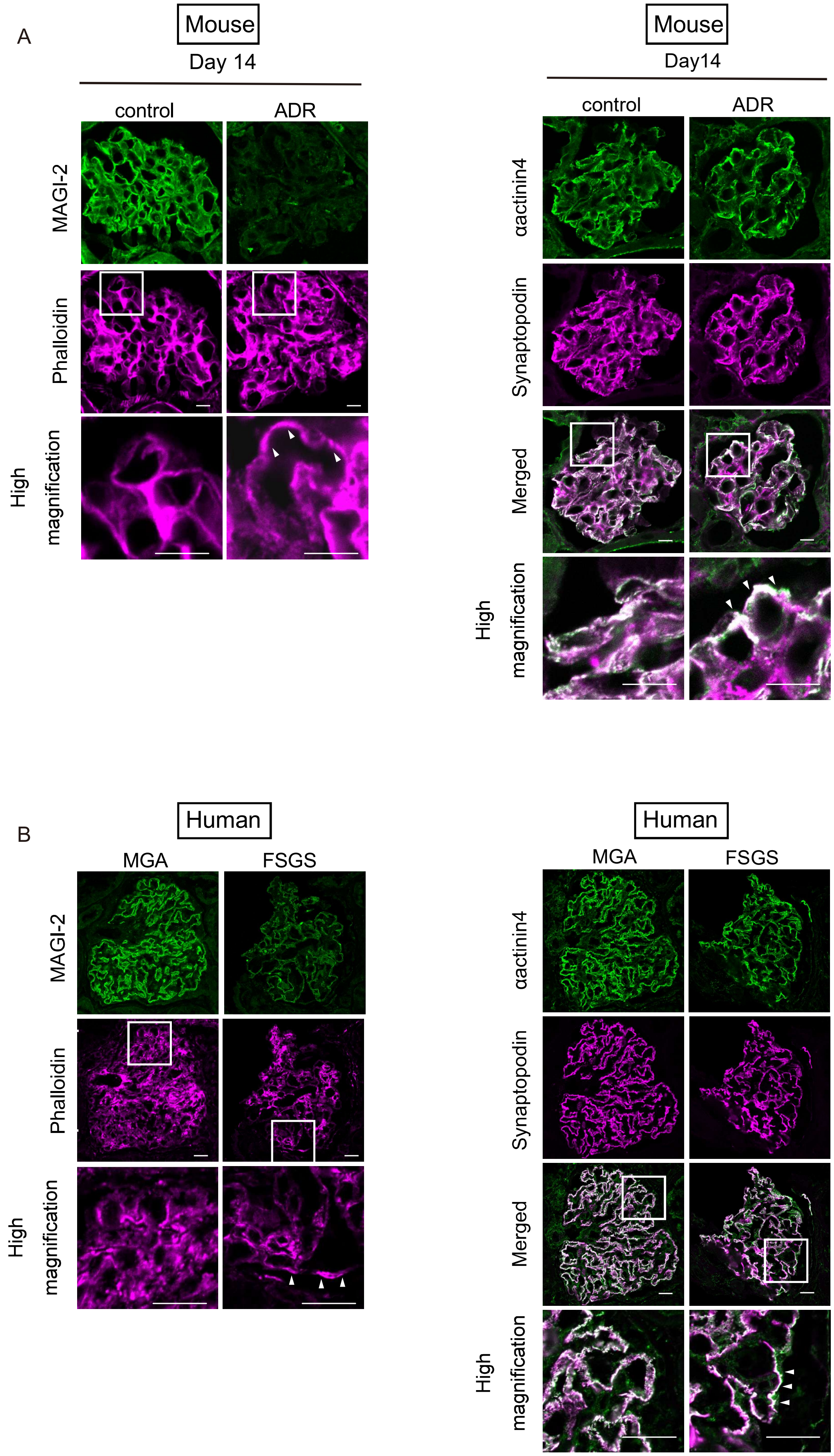

A
Mouse
Day 14
control
ADR
MAGI-2
Phalloidin
High magnification
Mouse
Day14
control
ADR
αactinin4
Synaptopodin
Merged
High magnification
B
Human
MGA
FSGS
MAGI-2
Phalloidin
High magnification
Human
MGA
FSGS
αactinin4
Synaptopodin
Merged
High magnification

Figure S5

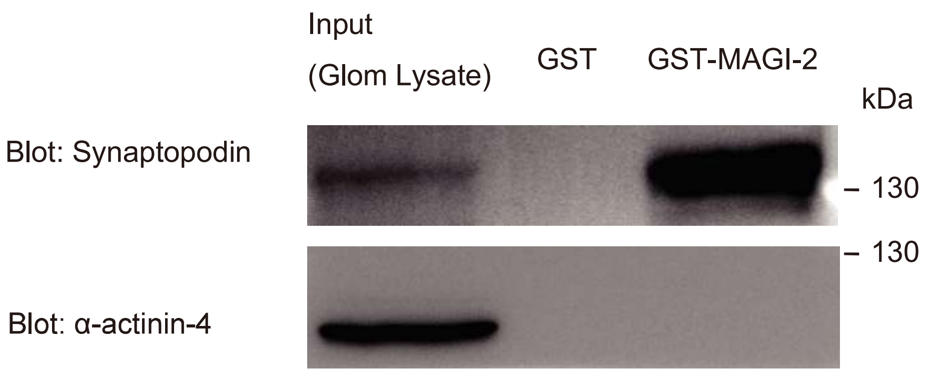
Input
(Glom Lysate)
GST
GST-MAGI-2
kDa
Blot: Synaptopodin
130
130
Blot: α-actinin-4

Figure S6

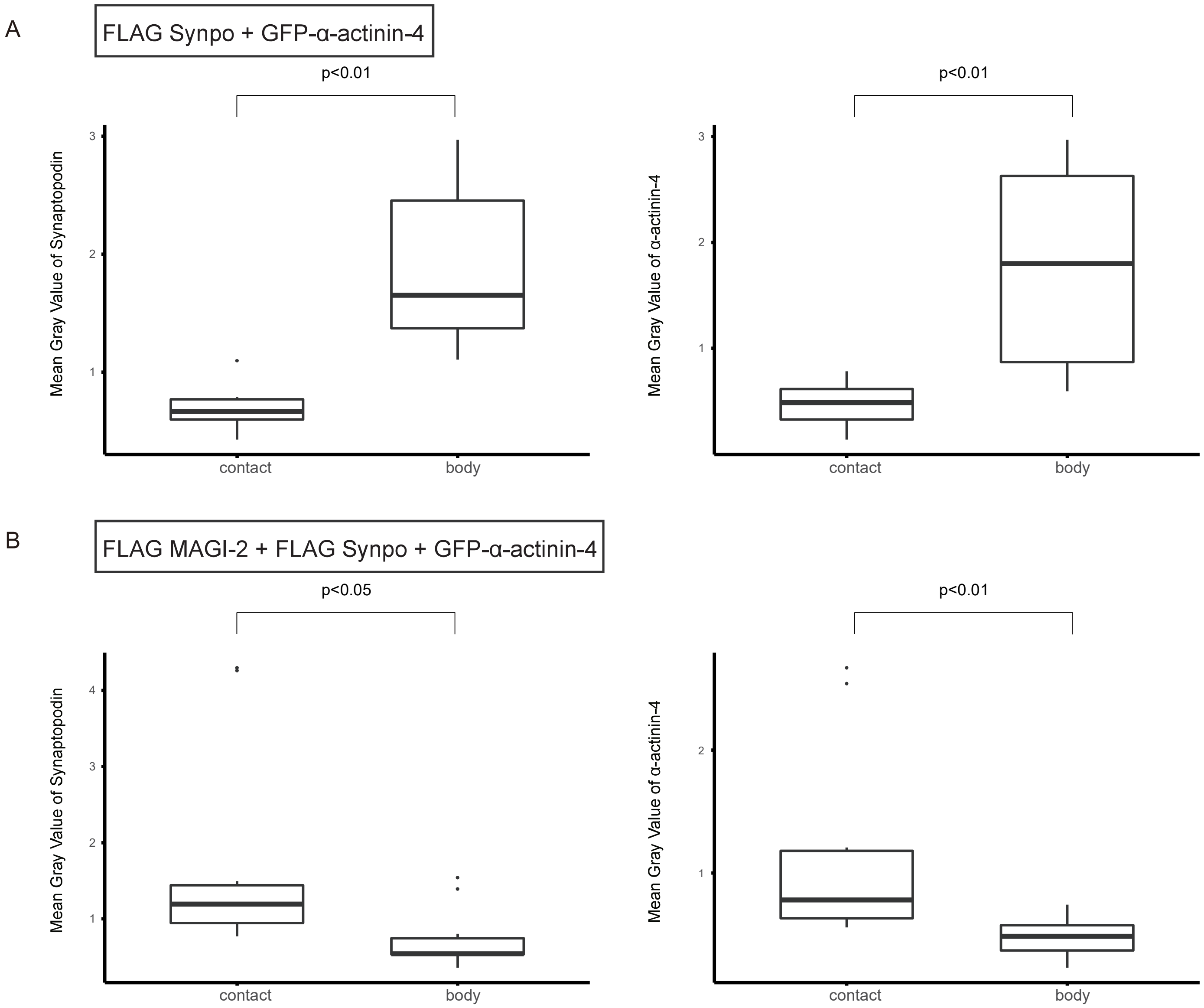
A
FLAG Synpo + GFP-α-actinin-4
p<0.01
Mean Gray Value of Synaptopodin
3
2
1
contact
body
p<0.01
Mean Gray Value of α-actinin-4
3
2
1
contact
body
B
FLAG MAGI-2 + FLAG Synpo + GFP-α-actinin-4
p<0.05
Mean Gray Value of Synaptopodin
4
3
2
1
contact
body
p<0.01
Mean Gray Value of α-actinin-4
2
1
contact
body

Figure S7

A

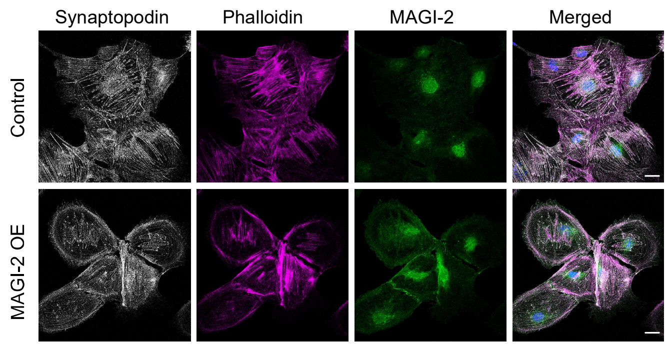

B

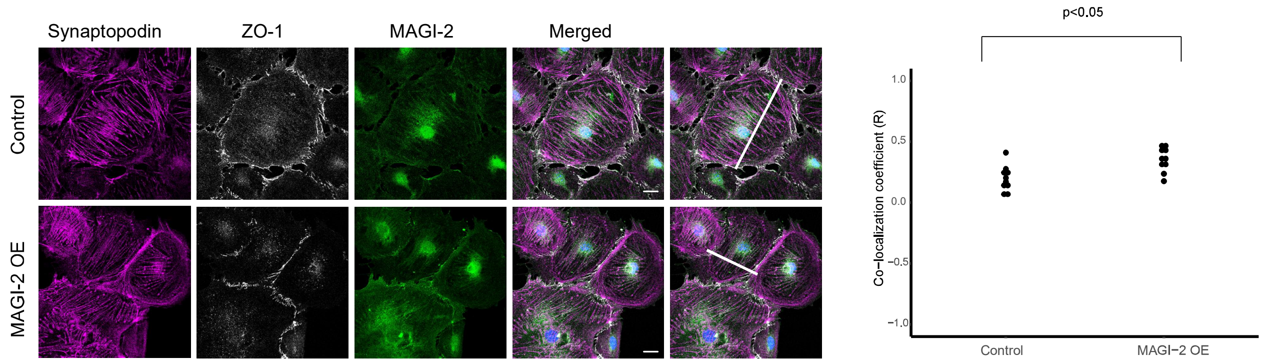

C

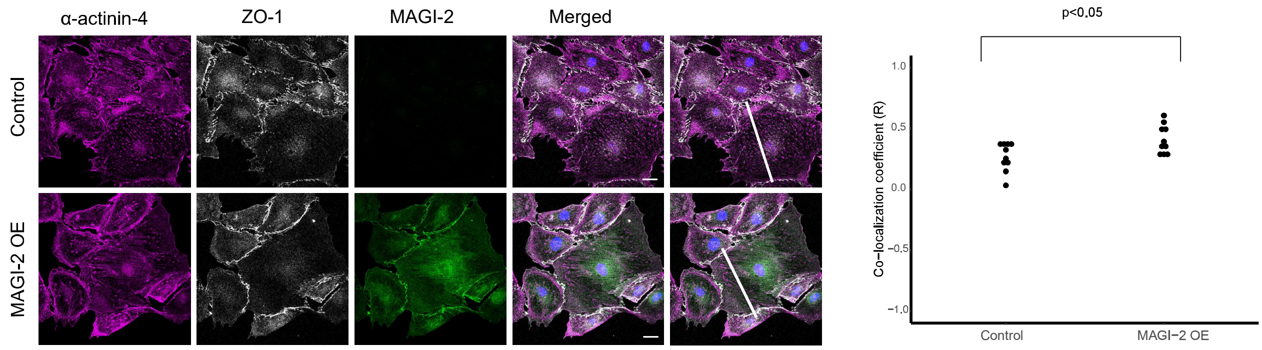

Figure S8

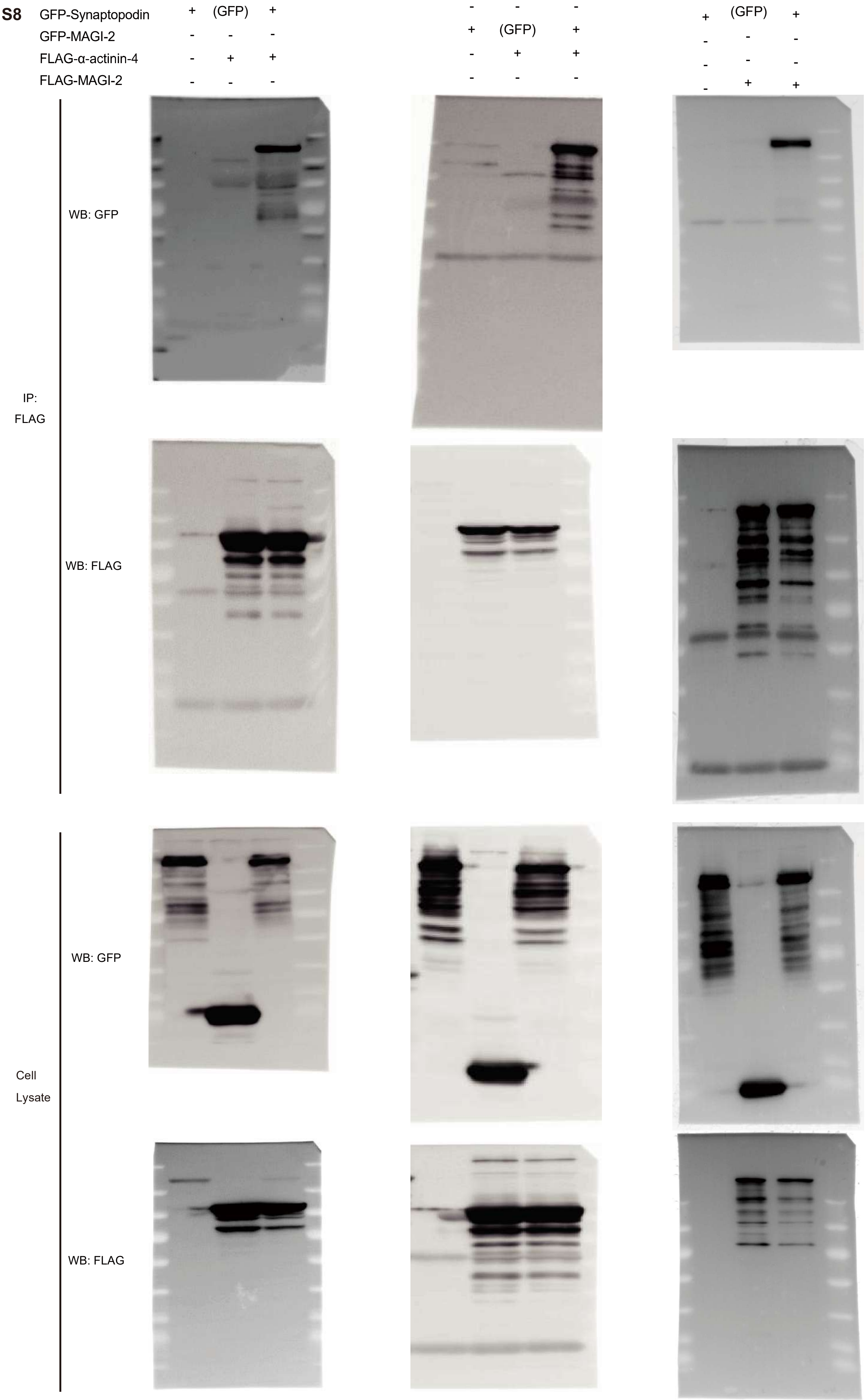
GFP-Synaptopodin + (GFP) +
GFP-MAGI-2 - - -
FLAG-α-actinin-4 - + +
FLAG-MAGI-2 - - -
- - -
+ (GFP) +
- + +
- - -
+ (GFP) +
- - -
- - -
- + +
IP:
FLAG
WB: GFP
WB: FLAG
Cell
Lysate
WB: GFP
WB: FLAG

Figure S9

GFP-Synaptopodin	+	+	+	+	+	+
GFP-MAGI-2	+	+	+	+	+	+
FLAG-α-actinin-4	-	+	-	+	-	+

Blot: Synaptopodin

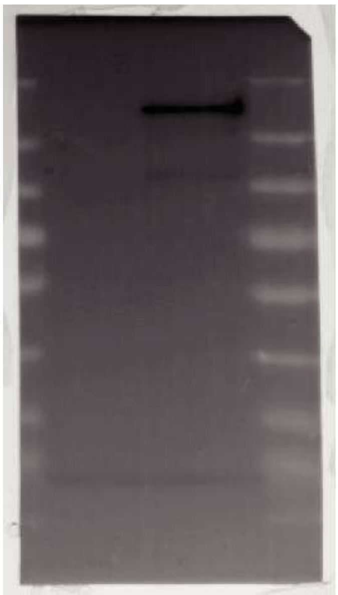

Blot: MAGI-2

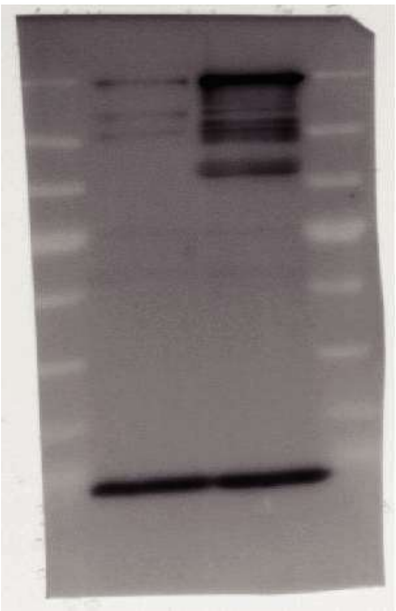

Blot: FLAG

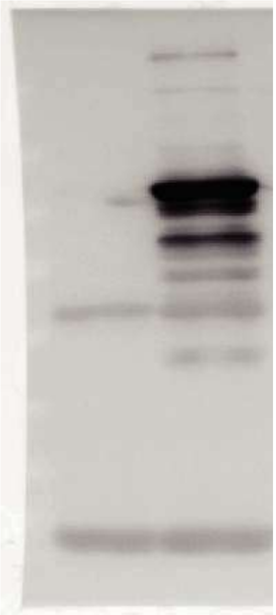

GFP-Synaptopodin	+	+	+	+	+	+
GFP-MAGI-2	+	+	+	+	+	+
FLAG-α-actinin-4	-	+	-	+	-	+

Blot: Synaptopodin

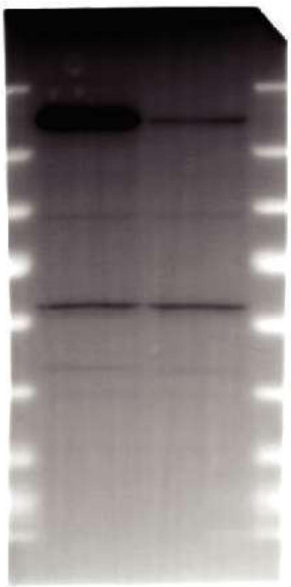

Blot: MAGI-2

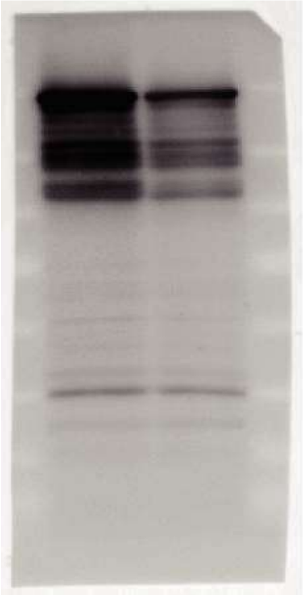

Blot: FLAG

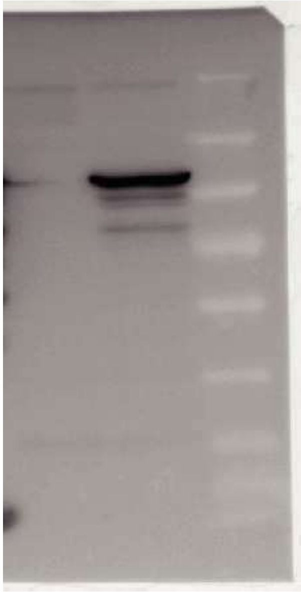

Supplemental Figure Legends

Figure S1. Triple immunofluorescence staining in wild-type mouse glomeruli to examine the localization of MAGI-2 and cytoskeletal proteins relative to nephrin.

(A) Representative images showing co-localization of MAGI-2 (green), α-actinin-4 (magenta), and nephrin (blue).

(B) MAGI-2 (green), synaptopodin (magenta), and nephrin (blue).

(C) Synaptopodin (green), α-actinin-4 (magenta), and nephrin (blue).

All images are from wild-type mouse kidneys and illustrate the spatial relationships among these proteins in podocyte foot processes**.** Bars=10μm

Figure S2. Altered localization of cytoskeletal and slit diaphragm proteins in inducible podocyte-specific MAGI-2 knockout mice and human glomerular diseases.

(A) Representative images of MAGI-2 (green) and Phalloidin (megenta) staining in control and MAGI-2 Ipd KO mice 1, 2, and 4 weeks after tamoxifen injection. The arrowheads indicate staining regions along the glomerular capillary walls, while the asterisks mark areas of staining in the mesangial regions. Bars=10μm (B)

Representative images of α-actinin-4 (green) and synaptopodin (magenta) staining in control and MAGI-2 Ipd KO mice 1, 2, and 4 weeks after tamoxifen injection. The white boxes indicate high magnification. The arrowheads indicate sharply stained areas. Bars=10μm

Immunofluorescence images represent analyses performed on kidney sections from four control and four podocyte-specific MAGI-2 knockout mice. For each mouse, three glomeruli were randomly selected and used for quantification. (n=20)

(C) Representative images of synaptopodin(green), podocalyxin(magenta), and nidogen(gray) staining in MGA and FSGS patients. Bars=10μm (D) The upper panel in D shows a higher magnification view of the area outlined by the white box in C. Quantification of the spatial relationships among green-, magenta-, and gray-labeled signals was performed. Regions where the three signals were aligned in parallel were selected, and fluorescence intensity profiles were obtained using profile plots drawn perpendicular to the gray signal. The distance from the peak green intensity to the peak gray intensity (b) was divided by the distance from the peak magenta intensity to the peak gray intensity (a). Twenty measurement points were randomly selected per group: from two patients with MGA and the normal renal cortex of one patient with renal cell carcinoma, and from three patients with FSGS. Bars = 1 μm.

† indicates MGA and normal portion of renal cell carcinoma.

MGA, Minor glomerular abnormalities, FSGS, Focal segmental glomerulosclerosis.

Figure S3. Quantification of MAGI-2 fluorescence intensity in mouse glomeruli.

Fluorescence intensity in the peripheral region of glomeruli was measured from MAGI-2 immunostained kidney sections. Three glomeruli were randomly selected per mouse (n = 4 per group). Background-subtracted mean fluorescence intensities were compared between control and MAGI-2 knockout mice using the Mann-Whitney U test.

Figure S4. Representative images of MAGI-2 and cytoskeletal protein localization in ADR nephropathy mice and human kidney samples.

(A) Immunofluorescence staining of MAGI-2 (green) and Phalloidin (magenta) in ADR nephropathy mice (left), and α-actinin-4 (green) and synaptopodin (magenta) in the same model (right). White squares indicate regions shown at higher magnification. The arrowheads indicate staining regions along the glomerular capillary walls.

(B) Immunofluorescence staining of MAGI-2 (green) and Phalloidin (magenta) in human kidney biopsy samples (left), and α-actinin-4 (green) and synaptopodin

(magenta) in the same samples (right). White squares indicate regions shown at higher magnification. The arrowheads indicate staining regions along the glomerular capillary walls. Bars=10μm

MGA, Minor glomerular abnormalities, FSGS, Focal segmental glomerulosclerosis.

Figure S5. GST pull-down assay using mouse glomerular lysates.

GST-tagged MAGI-2 fusion proteins or GST alone (negative control) were incubated with mouse glomerular lysates. Bound proteins were analyzed by western blotting using anti-synaptopodin and anti-α-actinin-4 antibodies. Glomerular lysates were included as input controls.

Figure S6. Quantification of fluorescence intensity in COS7 cells

Mean gray values (per μm) were calculated for cell body and cell contact regions and compared using the Wilcoxon rank-sum test.

(A) COS7 cells were transfected with FLAG-synaptopodin and GFP–α-actinin-4.

(B) COS7 cells were co-transfected with FLAG-MAGI-2, FLAG-synaptopodin, and

GFP–α-actinin-4. In (A) and (B), the left and right panels show the mean gray values of synaptopodin and α-actinin-4, respectively.

Synpo, Synaptopodin; contact, cell-cell contact; body, cell body

Figure S7. Synaptopodin and α-actinin-4 with the cell–cell contact marker ZO-1 in control and MAGI-2 OE podocytes.

Representative immunofluorescence images showing synaptopodin, α-actinin-4, ZO-1, and MAGI-2 staining in control and MAGI-2 OE podocytes. (A) Synaptopodin (gray), phalloidin (magenta), and MAGI-2 (green). (B) Synaptopodin (magenta), ZO-1 (gray), and MAGI-2 (green). (C) α-actinin-4 (magenta), GST (gray), and MAGI-2 (green). Bars=20μm

Figure S8. Full-length western blot membranes for co-immunoprecipitation (Figure 2A).

Full western blot membranes corresponding to the co-immunoprecipitation experiments shown in Figure 2A. HEK293T cells were transfected with GFP-tagged synaptopodin or MAGI-2 and FLAG-tagged MAGI-2 or α-actinin-4 as indicated. Lysates were subjected

to immunoprecipitation using FLAG beads, followed by immunoblotting with anti-FLAG and anti-GFP antibodies.

Figure S9. Full-length western blot membranes for co-immunoprecipitation (Figure 2B).

Full western blot membranes corresponding to the co-immunoprecipitation experiments shown in Figure 2B. HEK293T cells were transfected with GFP-tagged synaptopodin or MAGI-2 and FLAG-tagged α-actinin-4 as indicated. Cell lysates were subjected to immunoprecipitation using FLAG beads, followed by immunoblotting with anti-Synaptopodin, anti-MAGI-2, and anti-FLAG antibodies.
